# Decolonising the mindsets, attitudes and practices of the allopathic and indigenous health practitioners in postcolonial society: An exploratory approach in the management of patients

**DOI:** 10.4102/phcfm.v10i1.1518

**Published:** 2018-05-28

**Authors:** Simon M. Nemutandani, Stephen J. Hendricks, Mavis F. Mulaudzi

**Affiliations:** 1 School of Oral Health Sciences, Faculty of Health Sciences, University of the Witwatersrand, South Africa; 2 Albertina Sisulu Executive Leadership Programme in Health, Faculty of Health Sciences, University of Pretoria, South Africa; 3 School of Nursing, Faculty of Health Sciences, University of Pretoria, South Africa

## Abstract

**Background:**

The indigenous health care system continues in the postcolonial era to be perceived by antagonists as a threat to Western medicine. It has been associated with ‘witchcraft’, actively discouraged and repressed through official government prohibition laws. Despite that, human immunodeficiency virus and acquired immunodeficiency syndrome (HIV and AIDS) patients consult both allopathic and indigenous health practitioners.

**Aim:**

The study explored a collaboration model between allopathic and traditional health practitioners in the management of patients living with HIV and AIDS in postcolonial South Africa.

**Setting:**

We conducted six combined focus group discussions and four separate group discussions with each category of co-researchers.

**Methods:**

Combined and separate focus group discussions were conducted with community members, allopathic and indigenous health practitioners, applying the cyclical method in the decolonisation process. Their perceptions and experiences in the management of HIV and AIDS patients were explored, and finally decolonisation strategies suitable for collaboration in their context were identified.

**Results:**

The two health systems were rendering services to the same HIV and AIDS communities. Lack of communication created confusion. Collaboration was long overdue. A change in mindsets, attitudes and practices among practitioners was critical, with an acknowledgement that ‘neither health system is better than the other, but the two should be complementary, recognising that the culture and beliefs of patients influence their health-seeking behaviour’.

**Conclusion:**

Co-researchers were committed to working together in the fight against HIV and AIDS infections. Their model for collaboration addresses the challenges of patients’ secrecy, treatment overdose and the abandonment of antiretroviral treatment. Through the application of a decolonisation process, their mindsets, attitudes and practices towards each other were changed, enabling the joint development of a custom model for collaboration between allopathic health practitioners and indigenous health practitioners in the management of patients living with HIV and AIDS.

## Introduction

Acquired immunodeficiency syndrome (AIDS) and the causative agent, human immunodeficiency virus (HIV), have become one of the most serious health challenges that humanity is facing today.^1^ In 2016, there were global estimates of 37 million people living with HIV and AIDS.^2^ South Africa has the highest number of people living with HIV in the world (6.4 million).^1^ As more evidence supports that people living with HIV and AIDS consult both indigenous and allopathic health systems,^3,4,5,6,7^ an urgent need for a new and radical approach for collaboration among all health practitioners is required. The Euro-Western-centric approaches of ‘come join our rank’ type of collaboration encountered setbacks because of lack of trust and community ownership.^8,9,10^ Its often perceived to be aloof and focused on addressing allopathic problems. These approaches often do not recognise the existence of the acrimonious relationship between the two health systems, which is partly caused by centuries of colonisation.^11,12,13^

Thus, while the majority of South African patients rely on the current antiretroviral (ARV) drugs, which are wholly managed and controlled by the allopathic health system, some still prefer indigenous health medicine and practices to manage opportunistic infections.^4,5,6,7,14,15^ It appears that African communities have not completely adopted the Westernised approaches,^16,17^ despite being subjected to centuries of colonisation and dehumanisation of their tradition, beliefs and practices.^11^ At this current time, South Africans (including patients living with HIV and AIDS) are flexible and vacillate from allopathic to traditional practitioners and vice versa, seeking alternative advice, relief and treatment.^16,17^

The concept of developing a collaboration model was influenced by a call made by both allopathic health practitioners (AHPs) and indigenous health practitioners (IHPs) to respect the rights of patients to choose health practitioners of their preferences,^18^ and the overwhelming evidence that patients living with HIV and AIDS were consulting both systems.^3,4,5,6,7^

### Dual consultations and patients’ rights

Long before the AIDS pandemic, African communities had been consulting their IHPs for various physical, emotional and spiritual conditions.^19,20^ The authors of this article hold the view that patients do not belong to practitioners but are independent, with the opportunity to consult both the IHPs and AHPs.

The new government recognises the existing diversity of cultures, traditions and health beliefs,^21,22^ and promulgated the *Traditional Health Practitioners Act,* 2007 (Act No 22 of 2007), which accords citizens the right to consult IHPs.^23^ If patients are not satisfied with services, they have the right on request to be referred to a health provider of their choice for a second opinion.^18^

### Indigenous medicine in postcolonial society

During the colonial period, African culture, beliefs and practices were associated with witchcraft and evil powers.^23^ These indigenous conventions, including beliefs in the supernatural powers of the ancestors, were prohibited and smothered in terms of the *Witchcraft Suppression Act* 3 of 1957. The introduction of a Euro-Western health and belief system dominated and mitigated the destruction of the indigenous health system. It outlawed indigenous practices and associated their beliefs and culture with witchcraft and evil powers.^23^ The promulgation of the new Act marked an important apogee in the history of the postcolonial South Africa. The Act seeks to effect the declaration made by the World Health Organization (WHO) and the African Union (AU) that the traditional health system should be officially recognised and integrated into all aspects of health care provision.^23,24,25,26^

The exclusion of IHPs in the management of HIV and AIDS patients is mainly based on a monopolistic health system, which recognised allopathic health systems as the only practice. These dominances reinforced the stereotype which appears to suggest that patients belong to AHPs,^13^ and have no rights to seek alternative opinion and treatment other than what Western medicine prescribes.^27^ Such actions go against the provisions of the Patients and Human’ Rights Charter.^18^ It denies communities the power of making decisions for themselves.

A number of HIV and AIDS collaboration models have attempted to accommodate IHPs into the Western health system.^8,9,10^ These ‘come join our rank’ collaborations are dictated by, and are solely based on, terms and conditions determined by AHPs. Such models have mainly taken the form of organised training workshops where the IHPs were invited to ‘listen and learn from us’.^8,9,10^

### The emergence of indigenous epistemologies and methodologies

The impact of colonisation extended beyond politics and the economic life of indigenous communities. It disorientated and destabilised their psychosocial interactions with reality.^28,29,30,31^ There are growing perceptions that most colonised scientific scholars are unable to use their worldview to interrogate and interpret their world and environment, unless it meets the Western worldview.^30,31,32^ Whilst most of the colonised countries may have achieved political freedom from their erstwhile masters, the pervasive economic mindset persists, and liberation from scientific inclinations seems to evade indigenous scholars.^30,31^ It appears that they ignore indigenous epistemologies and subscribe to everything from the West,^28^ despite its limitations in African settings.^30,31,32^

As part of the rediscovery of self-determination of the indigenous people and recovery from the so-called ‘clutches’ of colonisation, a number of indigenous scholars have argued that the first thing that must be done is to decolonise the mindset and then to set an agenda for the indigenous-based research paradigm.^30,31,32^ This paradigm decolonises the indigenous mind by ‘re-centring’ indigenous values and cultural practices.^28^ It places the indigenous people and their agenda into dominant and mainstream discourses, which until recently have been relegated to the ‘side-lines’^29^ with marginalised indigenous communities being the subject of research conducted by Western researchers.^28^

It is further argued that postcolonial indigenous researchers should develop indigenous epistemologies and methodologies, which dismantle, deconstruct and decolonise the Euro-Western paradigms of thinking and of conducting research in indigenous communities^27,33,34,35^ Although the two health care systems operate side by side at different levels of sciences,^27,33^ that is, the theory of disease causation and management of disease, establishing collaboration between the two systems could have immense impact in the fight against HIV and AIDS in South Africa.^27,34^

The study aimed to explore through the decolonisation processes^30^ a model for collaboration between allopathic and traditional health practitioners to manage patients living with HIV and AIDS in postcolonial South Africa.

## Research methods and design

### Study design

This was a qualitative research method applying a phenomenological study design and using focus group discussions to explore an approach to manage HIV and AIDS among AHPs and IHPs in postcolonial South Africa.

### Settings

The study was conducted in all four districts of Limpopo Province in 2016. It involved IHPs, community leaders, patients living with HIV and AIDS and AHPs employed in public health facilities. Allopathic health practitioners include medical doctors, nurses and allied health workers.

### Study population and sampling strategy

The study population comprised IHPs, community leaders, HIV and AIDS patients and AHPs found in Limpopo Province in 2016.

Purposive sampling method was used to recruit participants from each district, ranging from allopathic and indigenous health practitioners, community leaders and patients living with HIV and AIDS in Limpopo Province, South Africa. There were six combined focus group discussions (CFGDs) conducted at the different health facilities (three hospitals and three community health centres) and four separate focus group discussions (SFGDs) with each category of participant in their work facilities (Table 1).

**TABLE 1:** List and description of participants.

Participants	Description	*n*
Allopathic health practitioners	Medical doctors	5
	HIV and AIDS coordinators	3
	Clinical psychologists	3
	Professional nurses	5
	Community health workers	5
	Pharmacists	3
	Social workers	2
Indigenous health practitioners	Herbalists	4
	Diviners	4
	Traditional surgeons	6
	Spiritualists	5
	Traditional birth attendants	3
Community leaders	Traditional chiefs	5
	Traditional council	8
	Headmen	2
	Hospital board members	3
HIV and AIDS patients	Consulting IHPs	4
	Consulting AHPs	4
	Consulting both sides	6

AHPs, allopathic health practitioners; IHPs, indigenous health practitioners; HIV and AIDS, human immunodeficiency virus and acquired immunodeficiency syndrome; *n*, number.

### Data collection method

**FIGURE 1:**
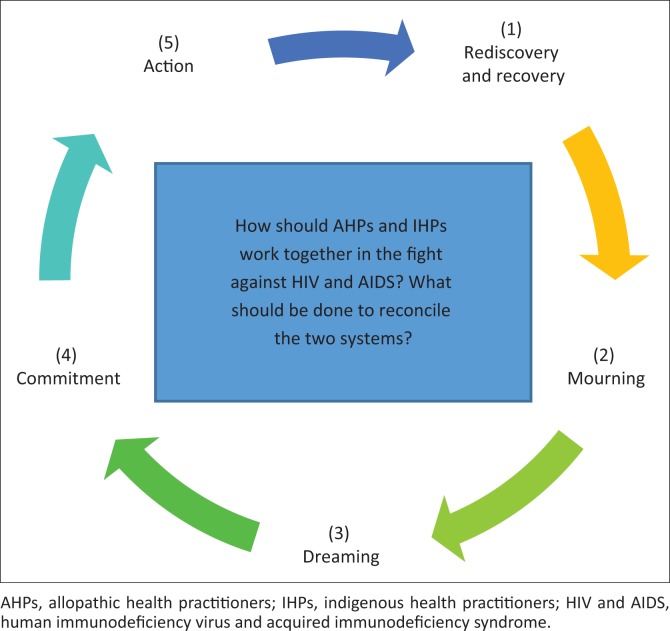
Cyclical pattern of decolonisation.

The interview guide consisted of three questions dealing with their opinion about coming together, the impact of colonisation and factors to consider for effective collaboration in postcolonial South Africa. The interview guide was translated into indigenous languages (Tshivenda and Xitsonga) by language specialists at the University of Venda. Two experienced co-researchers in conducting group discussions collected the data. The principal investigator led all the discussions and moderated the processes, whilst the other operated the audio recordings and collected field notes. The following cyclical pattern of the decolonisation processes was applied in both the combined and separate discussions (Figure 1). The revolving key questions across all steps were the following: how should AHPs and IHPs work together in the fight against HIV and AIDS? What should be done to reconcile these two health systems?

This approach of group discussions and collective decision-making empowers the community to seek solutions to identified problems. It fits well with the African philosophy and tradition of Ubuntu, ‘I am what I am because of who we all are’. It is an embodiment of community concept as it bestows respect on indigenous worldview and the intangibles of the community that reach beyond the allopathic concept of sciences. Such approach promotes social cohesion, ownership and sustainability of the jointly identified solution. Participants in the CFGDs were presented with excerpts from SFGDs and asked to discuss these. The aim was to gain further robustness of the findings and to retrieve a balanced approach to existing challenges.

Discussions were audio-recorded in Tshivenda and Xitsonga, transcribed verbatim and translated into English by a professional interpreter of both languages. Both transcription and translation were control-checked sentence by sentence by the external moderator.

### Data analysis

Two coders open-coded the transcripts paragraph by paragraph separately, jointly discussed and agreed on major code categories and harmonised them to have three final themes. These themes were presented at three different meetings of participants for critique and discussion to further refine analysis and conclusions. Final themes were endorsed during feedback discussion with participants, thus testing the transferability and trustworthiness of our findings.

### Ethical considerations

The ethical approval granted by the health research bodies (REC: 399/2013 and PMREC-54/2013) did not cover the requirements to enter the sciences, the world and the space in which IHPs operate. Signed informed consent was obtained from all the participants prior to participation. The leading researcher was further introduced to a ritual ceremony performed by IHPs to request the permission, guidance and approval from the ancestors.

## Results

The coming together of the two distinct health systems and the hosting of group discussions to explore an appropriate model for collaboration was a historic event. There had been no such platforms in the past, wherein common problems could be discussed. It brought different health practitioners and their patients together to collaborate on exploring the best approach to manage HIV and AIDS (see Table 1).

These meetings uniquely created an environment for the participants to share their opinions about their experiences. The analyses of exploratory meetings and discussions resulted in three main themes, (1) rediscovery and recovery of self-identity; (2) openness and honesty/mourning and dreaming; (3) commitment to change and action plan. The themes are introduced first, followed by subthemes that were substantiated by direct quotations from the participants.

### Rediscovery and recovery of self-identity

This was the first process of decolonisation. It provided an opportunity for participants to go through the process of interrogations, reflections and to discover their identity before colonisation. Their thought process enabled them to define their real world and the problems associated with lack of trust. Their views were categorised into three subthemes: (1) feeling of disbelief and shock, (2) recognition and acknowledgement and (3) opportunity to build trust and to establish relationships and collaboration.

### Disbelief and shock: *What is going on?*

The mere fact that IHPs were meeting with AHPs in full public view had considerable meaning for it was previously not possible. The IHPs felt a sense of being recognised and an acknowledgement as one of the role players in the South African health care system. There were feelings of shock and disbelief.

‘What is going on? Witches!! Are we now allowed to see patients in the hospitals? It looks like things are changing, ancestors will be happy.’ (F1, IHP, CFGD1)

What was unfolding in their lifetime appeared to be like a dream. The exclamation, ‘Witches!!’, could be the way in which they were expressing their years of suffering under apartheid and European laws as being evil and inhumane, dreadful and scary, comparable to ‘witches’. The meeting presented an opportunity for an outburst of emotions, with shouting as a way of rejecting the unwanted feeling of being treated with disrespect. The mourning process ensued, full of solace in the belief and affirmation that the ancestors were happy that finally recognition as health practitioners had been given.

It could also mean that the IHPs were reflecting on the past laws, namely, the *Witchcraft Act* of 1957, which referred to IHPs as ‘witches’, but now found themselves having a meeting with AHPs. This was unexpected and never anticipated, with the prospect of them entering the hospital premises with their traditional beads and wearing their regalia.

The feelings of excitement and shock were not the same for AHPs. Although they appreciated that the meeting was taking place, their opinions were focused on the lost opportunities to collaborate and the impact it had on patients: ‘I think it is long overdue; in fact, we’ve been bit slow … because our patients are one patient’ (M2, AHP, CFGD1)

### Recognition and acknowledgement

The meeting created a feeling of being accepted and also confirmed acknowledgement of the role played by IHPs. The following statement by an AHP appears to suggest that the change of mindsets and attitudes towards IHPs has been happening, although not often acknowledged in postcolonial South Africa:

‘…we know that several studies have been done which show that a huge number of our patients consult first with IHPs before coming to the hospital… Even very educated people, they consult with IHPs.’ (F4, AHP, CFGD3)

‘The Act is going to make it easy for us to work with you. I suppose it clearly states the roles and scope of your practices.’ (F3, AHP, CFGD4)

It is envisaged that the new Act will enable allopathic health practitioners to acknowledge and recognise that IHPs are part of the team of health workers. These views clearly indicate that some AHPs accept and acknowledge that IHPs have a role to play in the delivery of health care services in South Africa. In all our group discussions, this view was supported.

### Opportunity to build trust, establish relationship and collaboration

In the past, the relationship between AHPs and IHPs was always one of adversaries and competitors, mainly caused by years of colonisation and Europeanisation of the indigenous people. The process of decolonisation and the building of confidence and trust required honesty from all the participants. The existing differences were put aside in an effort to establish trust and create an environment for collaboration. The lack of communication between the two systems appeared to have placed AHPs under severe strain:

‘”ee”, maybe I should put it this way briefly, one can say, we are in the situation where we can no longer run away from rain because we are already wet, but what is important now is to look for a way forward.’ (M1, AHP, CFGD1)

The ‘rain’ symbolises a natural and powerful force which cannot be prevented. Therefore, another means for protection must be explored.

The meeting created an atmosphere of almost surreal astonishment as the two distinct health systems were brought together after a lengthy period of time of not talking to each other, let alone acknowledging the role that IHPs play in rural areas. Whilst there may be feelings of excitement about the meetings and optimism for collaborations, one should be cautious. High levels of suspicions and mistrust exist because of the lack of policy guiding collaboration, and the slow pace of recognising traditional medicine in South Africa. Such dominance, however, was not evident in our group discussions. The participants in our study expressed their opinions openly and committed themselves to working together to change negative attitudes, and to address the existing lack of trust between IHPs and AHPs.

### Mourning the disrespect of the indigenous medicine

The lamenting on the injustices is argued to be an important part of healing and preparing for the move to dreaming. The years of assault upon and damage done to the minds of indigenous people, their traditions, value and belief systems were evident in our discussions:

‘AHPs should first acknowledge that we are there, and accept us. Patients have the rights to consult both sides depending on their beliefs. We must first agree that we each have role to play in patients’ health, and both sides are competent. Unless you accept that, collaboration will not be possible.’ (M6, AHP, CFGD4)

Facial expressions, shaking of head and moving out of the room whilst IHPs were performing their opening ceremony were some of the actions which could be considered to be insensitive and disrespectful towards the belief and practices of others. The irony of it was that among those who were displaying signs of disrespect, there were AHPs with visible facial scars, evidence of the razor incisions normally performed by IHPs.

### Commitment to change and work together

Decolonisation processes appeared to have changed their attitudes, as they put their differences aside and focused seriously on respecting the rights and beliefs of patients to consult health practitioners of their choice. The following statement supports our assertion:

‘As we are seated around here, we are not merely representing ourselves as either IHPs or AHPs. It’s not about us, it is about the thousands of patients, represented by these members… We have no authority to tell a patient not to consult with IHPs neither to use traditional medicines’ …the critical and very important thing is that we should help the patient to survive, we should not contribute to the death of the patient.’ (M2, Traditional leader, CFGD1)

This comment by a traditional leader was the turning point. It appeared to unlock mindsets, which enabled change in the attitudes towards collaboration. The critical step was that of self-discovery by the participants who acknowledged that something had been wrong in their thinking.

‘…we have actually been colonised so much that we cannot even shake ourselves from the vision of the colonizers themselves. We are no longer able to see that we cannot continue functioning as separate individual healing systems.’ (F4, AHP, CFGD2)

The years of colonisation had not only destroyed the mind but also the very ability to escape the effect of colonisation. It affected the behaviour. As one AHP put it, they were no longer sure of what to do: ‘we need to be aware that we are confused’ (M4, AHP, SFGD2). Confused individuals usually experience problems with making decisions, and it may affect how those individuals perceive the world. Self-discovery had led the participants into realising that something was wrong, both in their actions and in their attitudes towards patients and towards each other. That deficiency needed to be fixed as part of changing the mindset.

‘We need to know…and it circulates around the issue of perceptions, perceptions, perceptions … we can’t be coconuts in dealing with collaboration issue. We need to meet, talk and talk and look at our challenges here and what should be the way forward and I think we need to unpack those issues…we need to talk to each other and meet so often, more often.’ (F3, AHP, CFGD4)

The perceptions and attitudes around collaborations with IHPs were now being brought to the front. The AHPs could avoid the issue no more. As stated in the opening remarks ‘it was long overdue’. There was readiness and convergence of ideas among the AHPs from different areas about the processes of collaboration, propelled by the understanding that ‘patients do not belong to us’ (F3, AHP, CFGD4). Furthermore, there was acceptance that as their health systems were different; there were differences in opinion regarding treatment modalities for patients living with HIV and AIDS. An IHP contextualised the problem by referring to it in a common phrase used among the communities:

‘”ndou mbili dzi tshi lwa, hu fa hatsi” it translates “When two elephants are fighting, the ground and grasses are destroyed”. This means when two health systems wage conflict with each other, patients will suffer.’ (M5, IHP, CFGD6)

The two elephants are compared to two health systems which have been fighting each other for years whilst patients suffered. There is no communication between them, yet they are treating the same patients, referred to as the ‘grasses and the ground’, suffering the consequences of overdose, drug interactions and unnecessary death at the end. The existing differences between traditional and allopathic health systems have affected how patients and communities adhere to ARV treatment. It was reported that patients were not disclosing that they were also consulting alternative health practitioners.

The remarks by a patient living with HIV and AIDS towards the end of the discussion were a plea to both practitioners to respect their beliefs and recognise their rights:

‘I heard both of you [*AHPs and IHPs*] stating that HIV/AIDS and TB patients do consult with you. I also heard the IHPs saying nurses tell our patients to stop taking their medicines. I think we must start there, that kind of approach to patients is wrong… for the patients to consult with you, it doesn’t mean you have the authority to stop them from exercising their beliefs: western or indigenous. No, you don’t have the right to do that…’ (M2, HIV/AIDS patient, CFGD5)

These comments appeared to change the mindset of the participants, that patients do not ‘belong’ to them. Patients are independent, and they should be allowed to consult whomsoever they wish, without fear of being blamed for consulting elsewhere. Observing the nodding of heads followed by ululations, we could conclude that after the robust discussions, these comments appeared to represent the feeling of almost all the participants. Unlike the Western practices of reaching decision by means of voting or consensus, nodding of heads, clapping of hands and ululations are common indigenous practices to confirm and support views.

The status quo and lack of collaboration among the health practitioners create confusion among the patients by separating the indigenous health system from allopathic health system.

‘As long as we separate these two healing systems, we are planting bad seed in patient’s minds…they find themselves confused and not knowing which healing system they should use. So, we plant this seed to people which make them think that if they use this healing system, they should not use the other.’ (F1, AHP, CFGD2)

The confusion was compared to a seed planted in the minds of patients. It grew with time and overpowered the rational mind with a colonised mind. The patient is then confused, and it is hoped that both the provider and the patient will recover from confusion to sober minds by following the same trajectory of changing the mindset but now through the ‘new seed’ of decolonisation of the mind process.

Noting that the confusion was caused by separating the two healing systems, the participants supported the concept of collaboration.

‘So, if we meet together like this, I am definitely sure that we shall manage to change people’s perceptions and mindset and they will know that these healing systems can complement one another. This is because patients consult with IHPs first there at their homes before they come to be part of those who consult with hospital treatment, isn’t it?’ (M4, AH, CFGD2)

## Discussion

The effect of years of colonisation and the indoctrination of African people to disown their ways of living and health practices was still evident in new democratic South Africa. The perception that traditional beliefs and practices belong to the dark ages and uncivilised societies^11^ appears to have resulted in a denial of the opportunity to accept IHPs.

The robust discussions sometimes raised emotions and invoked past injustices. However, honesty and openness ensured rational discussion with sober minds. The guiding principle remained: ‘it is not about us’ but about the patients and the HIV and AIDS pandemic which is not abating in South Africa.^36,37,38^ The increasing number of HIV and AIDS patients who are abandoning ARV treatment for traditional medicine^6,7,16,17^ is a strong case for the AHPs and IHPs to collaborate in the fight against the disease. The views of our participants were not different from the findings of a study conducted by Peu et al.^39^ in North West Province of South Africa.

Colonisation impacted adversely the relationship between IHPs and AHPs, and their management of patients living with HIV and AIDS. Participants resolved to address the status quo and lack of collaboration through decolonisation processes. The application of the cyclical decolonisation process resulted in a collective plan of action to manage their patients.

They demonstrated positive attitudes towards working together and showed appropriate respect, recognition and sensitivity required in collaboration. It was based on change of mindsets and attitudes towards each other’s sciences and practices. Furthermore, our participants committed themselves to work together to conduct co-training workshops to share knowledge and to learn from each other.

Despite the two health systems being affected differently, it was common cause that the concerns lay in the avoidable loss of life and the missed opportunities to work together to help the community. This process was crucial for establishing collaboration and building relationships in moving forward. The researchers recommend that a conducive non-judgemental accommodating platform should be created for collegial interactions between the two health systems. We recommend the incorporation of indigenous health system and its practices in the curriculum of health training institutions.

Although IHPs developed and agreed on the model for collaboration, they were representing their ancestors. Researchers could not verify that ancestors agreed with the developed model. It is only after the implementation wherein the approval or disapproval of collaboration may be postulated to be correct or not. The reluctance by health authorities to recognise indigenous health system as a science appeared to have limited the participation of AHPs.

## Conclusion

The participants appreciated the opportunity of the meeting whilst at the same time the legacy of colonisation was decried, recognising the adverse impact it has had on the indigenous communities and their culture, beliefs and practices. The application of decolonisation processes empowered and enabled participants to liberate themselves from the years of self-denial and hatred for their African identity. Neither health model was seen to be better than the other. They were recognised to be complementary to each other, and rendering alternative health care services in the best interest of patients and communities. The change of mindsets, attitudes and practices towards each other was central to the development of an appropriate model for collaboration in the management of patients living with HIV and AIDS in postcolonial South Africa.
